# 

**Published:** 1827-04

**Authors:** 


### MONTHLY LIST OF MEDICAL BOOKS.

[*No books can be entered on this List except those sent to us for the purpose; as, in the list hitherto transmitted, the names of works have frequently been given as published, which have not appeared for weeks, or even months, after.*]

A Clinical Lecture delivered to the Students of Surgery in the Royal Infirmary of Edinburgh, at the conclusion of the Winter Course for 1826-27. Edinburgh, February, 1827.

Psychological Speculations. Essay I. The Theological Department of Psychology, concerning Time, Space, Sense, &c. With a General Syllabus of Psychology. By the Spirit of the Blue Mountains.

Reply to the "Additional Strictures" contained in the first Number of the Quarterly Medical Review, on the Principles of Dental Surgery, exhibiting a New Method of Treating the Diseases of the Teeth and Gums, especially calculated to promote their Health and Beauty, &c. By LEONARD KOECKER, Surgeon-Dentist, Doctor in Medicine and Surgery, &c. &c. &c.

Medical Botany, No. III. Containing Hyoscyamus Niger, Phellandrum Aquaticum, Helleborus Niger, Lactuca Virora.—The Plates are beautifully executed.

A Series of Engravings, intended to illustrate the Structure of the Brain and Spinal Chord in Man. By HERBERT MAYO, Surgeon, and Lecturer on Anatomy. *** We can vouch for the accuracy of these as representations of the parts, having compared several of them with the preparations from which they have been taken. As engravings, they are beautiful specimens of the art; and the whole is highly creditable to Mr. Mayo.

---

### METEOROLOGICAL JOURNAL,

### NOTICES.

*Communications have been received from* Dr. Burder, Mr. Cox, Dr. Gregory, Dr. Webster, Mr. Boyle, Mr. Russell, Mr. Kingsley, Mr. Wallace, Dr. Mac Andrew, *and* Mr. Hamilton.

Mr. T.'s *Case of Retenitis does not appear to us of sufficient interest for publication.*

*We regret we cannot insert the Communication with which we have been favoured by* Mr. K——y. *The subject is not devoid of interest, but the cases are related much too diffusely for publication.*

*The present Number had unfortunately been made up before* Mr. Wallace's *letter arrived. We shall give it in our next.*

N.B.—*We have recently received several pamphlets, and other large packets, by post. We must suggest to Correspondents, that we cannot receive any Communications, the carriage of which is not paid.*

